# Copy Number Variation among Resistance Genes Analogues in *Brassica napus*

**DOI:** 10.3390/genes13112037

**Published:** 2022-11-04

**Authors:** Aria Dolatabadian, Yuxuan Yuan, Philipp Emanuel Bayer, Jakob Petereit, Anita Severn-Ellis, Soodeh Tirnaz, Dhwani Patel, David Edwards, Jacqueline Batley

**Affiliations:** 1 School of Biological Sciences, University of Western Australia, Perth, WA 6009, Australia; 2 School of Life Sciences, The Chinese University of Hong Kong, Hong Kong, China

**Keywords:** canola, disease resistance, genomic structural variation, oilseed crops

## Abstract

Copy number variations (CNVs) are defined as deletions, duplications and insertions among individuals of a species. There is growing evidence that CNV is a major factor underlining various autoimmune disorders and diseases in humans; however, in plants, especially oilseed crops, the role of CNVs in disease resistance is not well studied. Here, we investigate the genome-wide diversity and genetic properties of CNVs in resistance gene analogues (RGAs) across eight *Brassica napus* lines. A total of 1137 CNV events (704 deletions and 433 duplications) were detected across 563 RGAs. The results show CNVs are more likely to occur across clustered RGAs compared to singletons. In addition, 112 RGAs were linked to a blackleg resistance QTL, of which 25 were affected by CNV. Overall, we show that the presence and abundance of CNVs differ between lines, suggesting that in *B. napus*, the distribution of CNVs depends on genetic background. Our findings advance the understanding of CNV as an important type of genomic structural variation in *B. napus* and provide a resource to support breeding of advanced canola lines.

## 1. Introduction

Current advances in high-throughput sequencing techniques have simplified and accelerated genomic studies and made it easier to reveal the genetic diversity among different individuals. Genome-wide DNA variations have traditionally included single-nucleotide polymorphisms (SNPs) and insertion/deletions (InDels). In recent years, pangenomes representing the entire genetic content of a species, have become popular as a tool to study genomic variants without reference bias [1,2]. There are now published pangenomes across plants such as *B. napus* [3,4,5], *B. oleracea* [6,7], wheat [8], *Amborella* [9], pigeon pea [10], sesame [11], rice [12,13], soybean [14,15,16], and banana [17]. These pangenomes facilitate the identification of copy number variation (CNV) [18,19] and highlight the importance of CNVs in the evolution and functionality of genes related to crop agronomic traits [3].

A CNV is defined as a genomic sequence variant larger than 50 bp [20] to over several Mbp in size [21], consisting of deletions, insertions, duplications or translocations [22]. Gene CNVs occur due to errors in homologous recombination events [23] and are observed in many organisms resulting in dozens to hundreds of differences in their number of functional genes [24].

CNVs affect gene and protein expression levels and eventually influence the phenotype [25] and evolutionary adaptation [26]. There are increasing reports associating CNV with major traits in different crop species, but the extent and role of CNVs in plants are not yet fully understood [27]. CNVs may have broad implications for model organism research, evolutionary biology, and genomics-assisted breeding approaches to improve crop adaptation and yield [28,29].

Since CNVs are ubiquitous and encompass more nucleotides per genome than the total number of SNPs [21,30], more attention has recently been paid to their role. There are a growing number of investigations in plant species such as maize [31,32,33], *Arabidopsis* [28,34,35], rice [36,37], wheat [38,39], barley [40,41,42], banana [43], tomato [44], and soybean [45,46], suggesting that genes affected by CNVs are associated with agronomically important traits.

CNVs have been identified as plant disease defense genes in various species [45,47,48,49,50,51,52]. For example, Hu et al. (2018) identified an association between CNV of *rp1* and resistance to Goss’s Wilt of maize [52]. Another example is soybean cyst nematode resistance, which is driven by CNV of the locus *Rhg1* increasing expression of a set of genes [45,53]. In canola, genes located within QTL linked to resistance to *Verticillium longisporum* are affected by CNV [54] and local duplication of a TNL gene is likely to be involved with clubroot resistance in *B. napus* cv. Tosca [55]

Canola (*Brassica napus* L. AACC, 2*n* = 38) is an allopolyploid that originated from natural hybridization events between the two diploid species *B. rapa* (AA, 2*n* = 20) and *B. oleracea* (CC, 2*n* = 18) [56]. Canola production is affected by several important diseases, of which blackleg, caused by the fungus *Leptosphaeria maculans*, is the most important disease. Although resistant canola cultivars have been developed through targeted introduction of resistance genes by breeding, yield losses still occur due to resistance breakdown. This breakdown highlights the importance of identifying and characterizing novel resistance genes. Resistance gene analogues (RGAs) are the most important component of the host resistance mechanism [57]. Classes of RGAs include nucleotide-binding site leucine-rich repeats (NLR), receptor-like proteins (RLPs) and receptor-like kinases (RLKs). CNV events may lead to additional copies of resistance genes, suggesting that CNV can be beneficial and a mechanism driving resistance [58].

Although *B. napus* is a model species for studying phenomena such as polyploidy [59], genomic rearrangements [60,61], its resistance at the cotyledon stage to blackleg is a typical example of qualitative resistance involving RGAs [62]. However, there are still few studies of CNV within the *B. napus* genome [29,54] and their effect on qualitative resistance. The present study is the first genome-wide analysis of copy number variation across RGAs among various morphotypes of *B. napus*. As RGAs are responsible for qualitative resistance, the CNV events were also investigated across blackleg resistance-linked regions. In this study, we investigated and detected deletions and duplication events, as these types of CNV are likely more associated with disease resistance or susceptibility. Our analysis provides new insight into CNVs in canola cultivars and will help identify the role of CNV in resistance.

## 2. Materials and Methods

### 2.1. Plant Materials

Eight winter type *B. napus* lines were used in this study. The selected lines have various important agronomic characteristics. All are resistant to blackleg, but may carry different blackleg resistance genes. Ascona (breeder: SW Seed, New South Wales, Australia), Pirola (breeder: KWS, New South Wales, Australia), Milena (breeder: KWS, New South Wales, Australia) and Pacific (breeder: Limagrain-Nickerson, Lincolnshire, UK) are canola quality lines and widely cultivated [63]. English Giant (breeder: Afrigro Seed Company, Oudtshoorn, South Africa) is one of the most popular lines in Zimbabwe (favourable because of its hardness) [64], Tina and Wilhelmsburger (type: swede) are resistant to the pathogen *Plasmodiophora brassicae* [65,66] and HANSEN × GASPARD DH LINE (breeder: KWS, New South Wales, Australia) is partially resistant to *Sclerotinia sclerotiorum* [67]. Tina was released in the early 1980s [65], Wilhelmsburger [68] and English Giant [69] were used in the 1960s and the rest of the varieties were available no later than 2010 [63,70].

### 2.2. DNA Extraction and Quantification

Genomic DNA was extracted and purified from fresh young leaves of all *B. napus* lines using a Qiagen (Qiagen, Germany) DNAeasy kit following the manufacturer’s instructions. Total DNA was quantified using the Qubit 3.0 Fluorometer with the Qubit dsDNA HS Assay Kit (Invitrogen, Waltham, MA, USA) following the manufacturer’s instructions. After quantification, each DNA sample was diluted to 10 ng μL-1.

### 2.3. Construction of Genomic DNA Libraries and Sequencing

Libraries were constructed using the Illumina TruSeq^®^ Nano DNA Library Prep kit (Illumina, California, CA, USA) according to the manufacturer’s instructions. The libraries were quantified using a Qubit, and the quality was assessed using a LabChip (GX Touch 24, PerkinElmer, Waltham, MA, USA). The concentration of the library was adjusted to 10 nM. The whole genome was sequenced pair end (150 bp) using a HiSeq X Ten sequencing platform at the Garvan Institute of Medical Research (Sydney, NSW, Australia).

### 2.4. Sequencing Data Processing and Read Alignments

Trimmomatic v0.36 [71] was used to trim adapters and remove low quality reads shorter than 150 bp. The reads from each line were aligned to the *B. napus* Darmor-bzh v9 reference genome [5] with default settings using SpeedSeq v0.1.2 [72] and BWA v0.7.10 [73]. The resulting alignment files were sorted and indexed using SpeedSeq. SAMBAMBA v0.5.9 [74] was used to mark duplicates.

For phylogeny analysis, SNP calling was performed using bcftools and only the biallelic SNPs were kept. A Neighbour Joining tree was made using vcfkit.

### 2.5. CNV Calling

CNVs were called using CNVnator v0.3.3 [75]. Different bin sizes were used to ensure the standard deviation of read depth signal was in the range 4 to 5 as recommended. To reduce false-positive calls, the CNVnator result was filtered by removing CNVs with an e-value ≥ 0.05 and q0 value ≥ 0.5 using BCFtools v1.5 [76]. CNVs overlapping at least 50% with gap regions (N) were removed using BEDTools v2.25.0 [77] intersect (parameters: -f 0.50 -r -v). After filtering, RGAs were associated with CNVs if they overlapped for more than 50% of their length using BEDTools v2.25.0 [77] intersect (parameters: -f 0.50).

### 2.6. RGA Prediction and Physical Clustering

The RGAugury pipeline (v 2017-10-21) [78] was used to automate RGA (NLR, RLK, and RLP) prediction in the *B. napus* Darmor-bzh NRGene v9 annotation. RGA candidates were classified into subclasses based on the presence or absence of specific domains. The NLR candidates were divided into classes based on domain presence. Proteins carrying only an NB-ARC domain were classified as NBS, proteins carrying TIR, NB-ARC, and Leucine-Rich-Repeat (LRR) domains were classified as TNLs, or TN if the LRR domain was missing. Proteins carrying Coiled-Coils, NB-ARC, and LRR domains were classified as CNLs, or CN if the LRR domain was missing, or NL if the Coiled-Coils domain was missing. Proteins carrying a TIR domain with additionally unknown domains were classified as TX. Other combinations (e.g., CNL + RPW8) were classified as OTHER. RGAs were joined into physical clusters if they were located within ±10 genes of each other.

### 2.7. QTL and Genomic Data Representation

Known blackleg resistance-linked QTL were collected from the literature [79,80,81,82,83] and the sequences of the markers, genes and primer pairs were downloaded. BLAST [84] was used to assign positions for the forward and reverse primer sequences. Circos plots were generated using Circa (http://omgenomics.com/circa accessed in 2019) and Circos (http://circos.ca/ accessed in 2019).

## 3. Results

### 3.1. CNV Analysis

**Figure 1:**
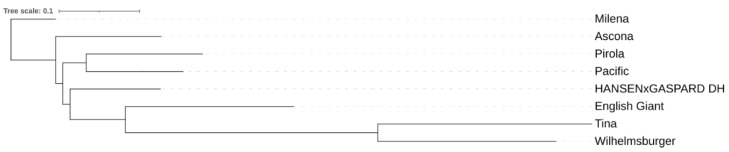
Phylogeny analysis of eight winter type and blackleg resistant *B. napus* cultivars; Ascona, English Giant, Hansen × Gaspard, Milena, Pacific, Pirola, Tina and Wilhelmsburger.

To investigate the role of CNVs in RGA-diversity in *B. napus,* we generated whole-genome sequencing data to search for CNVs among RGAs of eight *B. napus* morphotypes; Ascona, English Giant, Hansen × Gaspard, Milena, Pacific, Pirola, Tina and Wilhelmsburger. While all lines are winter type and blackleg resistant, they are of interest for other characteristics including canola quality (widely cultivated) and resistance to diseases other than blackleg. The phylogeny analysis of lines is shown in Figure 1.

**Figure 2:**
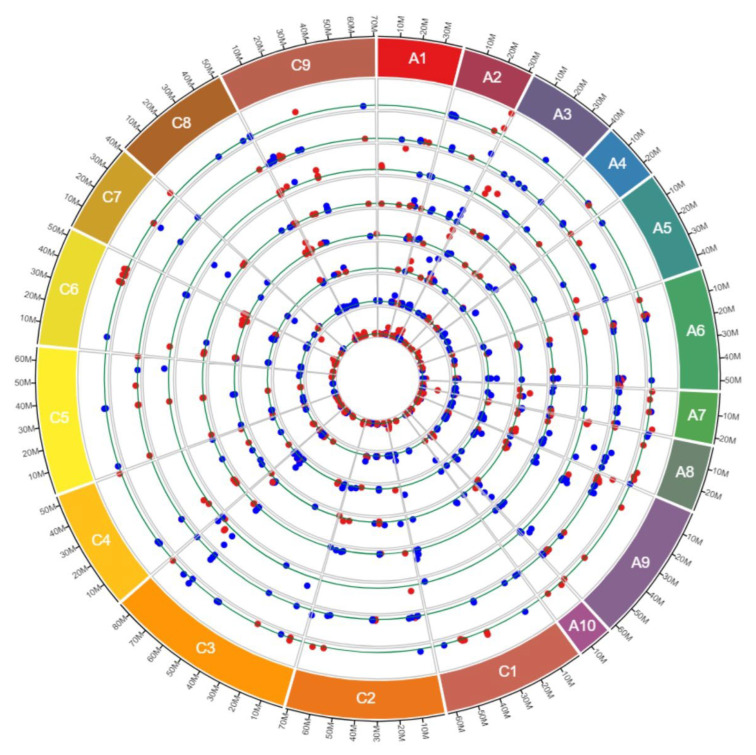
The distribution and size of CNV events in RGAs (red and blue dots represents deletions and duplications, respectively) across the chromosomes of eight winter type and blackleg resistant *B. napus* cultivars. The tracks from outer to inner show chromosomes, Ascona, English Giant, Hansen × Gaspard, Milena, Pacific, Pirola, Tina and Wilhelmsburger. The green line shows the 1000 bp threshold. Ax and Cx in the outer coloured boxes are presenting chromosomes number.

**Figure 3:**
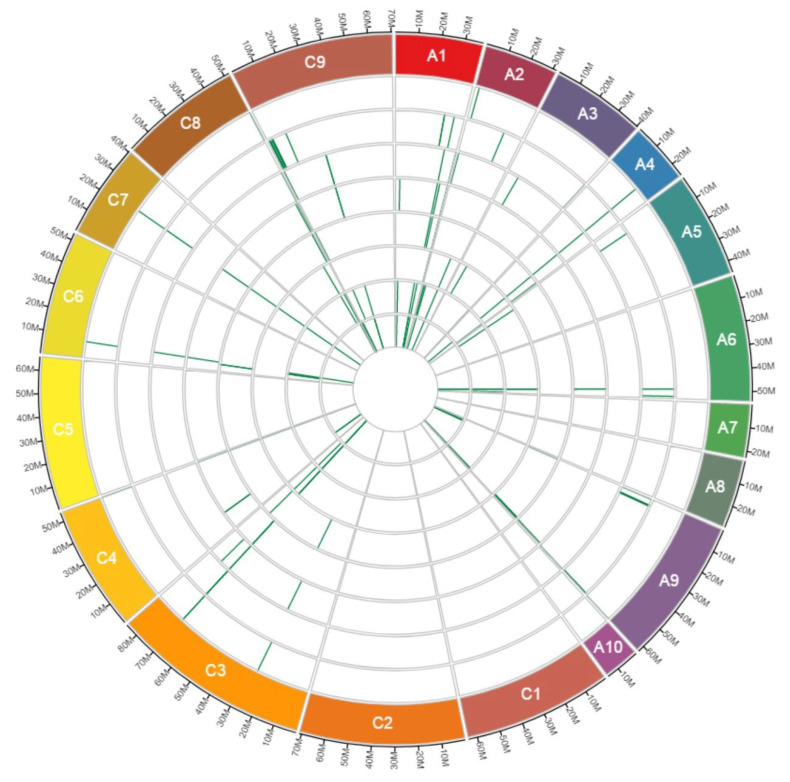
The position of “both deletion and duplication” events in RGAs across the chromosomes of eight winter type and blackleg resistant *B. napus* cultivars. No “both deletion and duplication” events were detected on chromosomes A07, A08, A10, C01, C02 and C05. The tracks from outer to inner show chromosomes, Ascona, English Giant, Hansen × Gaspard, Milena, Pacific, Pirola, Tina and Wilhelmsburger. Ax and Cx in the coloured boxes are showing chromosomes number.

Our study detected a total of 1,137 CNV events (deletions and duplications) with a total size of 3.74 Mbp across 563 RGAs. On average, we found 142 CNVs per cultivar, representing an average of 3.29 kb across the eight cultivars. Out of the 1,137 CNV events, 704 (61.92%, 2.58 Mbp) were deletions and 433 (38.08%, 1.16 Mbp) were duplications, with an average of 88 and 54 events, respectively (Table 1). We found 1.6× more deletion than duplication events, and on average deletions were larger (3.67 kb) than duplications (2.66 kb). The largest deletion and duplication percentages were found in the cultivars Tina (68.20%) and Pacific (50%), respectively (Figure 2 and Table 1). We identified 188 CNV events (16.53%) that showed deletion in one cultivar, but duplication in another, which are termed as “both deletion and duplication”. These “both deletion and duplication” events were detected on all chromosomes except A07, A08, A10, C01, C02 and C05 (Figure 3 and Figure S1).

Based on the number of CNV events detected in each cultivar, Hansen × Gaspard with 26.98% and Pirola with 11.86% contained the largest and lowest percentages of these “both deletion and duplication” CNV events, respectively, (Table 1).

### 3.2. Distribution along Chromosomes and Sub-Genomes

**Figure 4:**
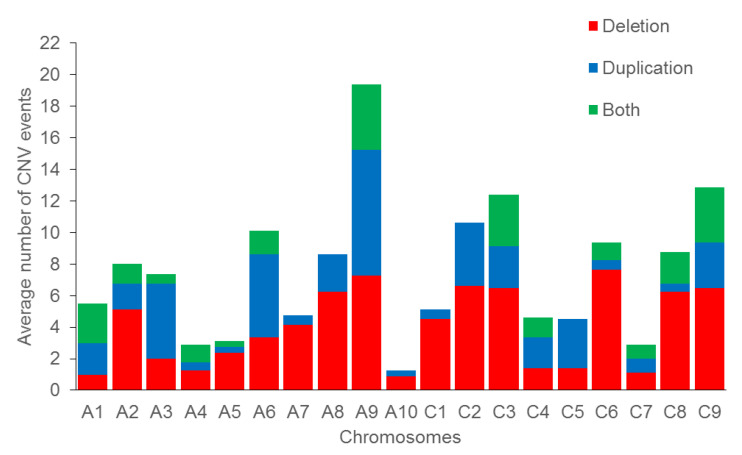
The average number of CNV events (i.e., deletion, duplication and both deletion and duplication) per chromosome of eight winter type and blackleg resistant B. napus lines. Each colour (bin) represents a different CNV events and the bars show the average number of CNVs falling into each event bin. Both: Where both deletion and duplication events occurred.

The average number of CNV events per chromosome ranged from 19.37 on chromosome A09 to 1.25 on chromosome A10 (Figure 4 and Table S1). In cases where both deletion and duplication events were observed, the largest deletion and duplication percentages (in relation to the total number of CNV events on each chromosome) were found on chromosomes C08 (30 deletions out of 31 CNVs; 96.77%) and A03 (12 duplications out of 14 CNVs; 85.71%) in the cultivars Tina and Pirola, respectively (Figure 4 and Table S1).

Across all cultivars CNVs showed an even distribution over the A sub-genome (568 CNVs) and the C sub-genome (569 CNVs), yet when separated by cultivar, there were more CNVs in the A sub-genome than in the C sub-genome in English Giant (A:96, C:60), Hansen × Gaspard (A:37, C:26), Milena (A:55, C:50) and Pacific (A:62, C:54), and more CNVs in the C sub-genome than in the A sub-genome in Ascona (A:35, C:50), Pirola (A:56, C:62), Tina (A:114, C:125) and Wilhelmsburger (A:113, C:142) (Table S1). Overall, deletions were more abundant than duplications in both the A (317 vs. 251) and C (387 vs. 182) sub-genomes (Table S1).

**Figure 5:**
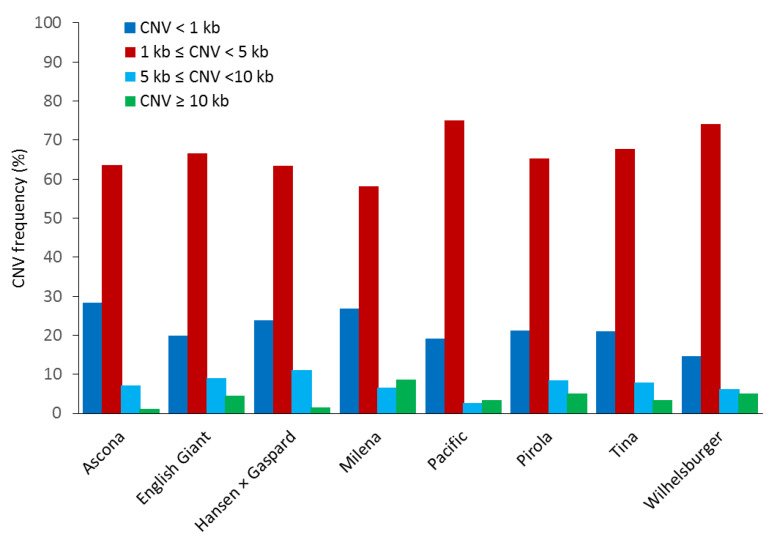
Size range distribution of CNVs in eight winter type and blackleg resistant B. napus lines. Each colour (bin) represents a different range of CNV lengths and the bars show the percentage of CNVs falling into each size bin.

Out of the 1,137 CNV events, 905 CNVs (79.59%) were found to be larger than 1 kb (Table S2). The average size of the CNVs identified varied from 1.91 kb in Ascona to 4.90 kb in Milena, with an average size of 3.29 kb across the eight cultivars (Table S2). In all the cultivars, except for Hansen x Gaspard, deletions were larger than duplications (Table S2 and Figure 2). The size distributions of observed CNVs were also very similar between the eight cultivars. Only Milena and Pacific had more CNVs larger than10 kb than CNVs smaller than 10 kb but larger than 5 kb (Figure 2 and Figure 5).

### 3.3. CNVs across RGAs

**Figure 6:**
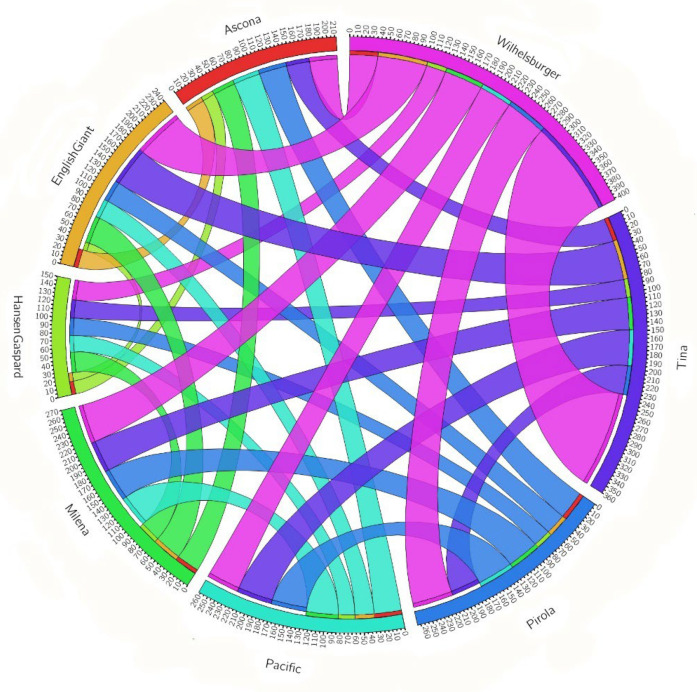
Circos plots showing the number of RGAs with the same CNV events among eight winter type and blackleg resistant *B. napus* cultivars. Each ribbon shows number of RGAs with CNV in common, the wider ribbon, the highest number of RGAs with the same CNV.

We identified 563 RGAs overlapping with CNVs including 164 NLR, 319 RLK and 80 RLP genes. The largest classes of RGAs affected by CNV across the eight cultivars were RLK and RLP (on average 50.21% RLKs and 16.86% RLPs in each cultivar) (Table S3). Among the NLR sub-families, NL and TNL were the most abundant RGAs affected by CNV events (Table S3). Out of 563 RGAs, 310, 196 and 57 genes showed deletion, duplication and “both deletion and duplication”, respectively (Table S4). No “both deletion and duplication” events were detected on chromosomes A07, A08, A10, C01, C02 and C05 (Figure 3). Across all eight cultivars, multiple RGAs overlapping CNV were shared between two or more cultivars (Table 2). The highest and lowest two cultivar overlap was 126 between Tina and Wilhemsburger, and 11 between English Giant and Hansen × Gaspard (Table 2). The number of RGAs with CNV in common between the cultivars is depicted in Table 2 and Figure 6. Out of 563 RGAs showing CNV, 262 (46.54%) were detected only in one cultivar and two (0.36%) were shared in all cultivars (Table 3).

### 3.4. Gene-Physical Clustering

Out of 1768 RGAs previously identified in the *B. napus* Darmor-bzh NRGene v9 annotation, 793 RGAs were clustered in 306 physical clusters, of which 284 RGAs (35.81%) (121 NLRR, 110 RLK and 53 RLP) were affected by CNV (180 deletions, 75 duplications and 29 both) (Tables S4 and S5). In addition, there were 975 singleton RGAs, of which 279 RGAs (28.61%) (43 NLR, 209 RLK and 27 RLP) were affected by CNV (130 deletions, 121 duplications and 28 both) (Table S4). The distribution and number of the singletons and clustered resistance genes affected by CNV across the chromosomes are presented in Table S5.

### 3.5. Investigating of RGAs Affected by CNV Events across Known Genomic Regions for Blackleg Resistance Genes

The RGA positions were compared with known regions for blackleg resistance to identify possible candidate genes affected by CNV. Positions were predicted for 14 markers from genetic mapping of seven loci: LepR1 (A02), LepR2 (A10), Rlm1, Rlm3, Rlm4, Rlm7 and Rlm9 (A07) in the Darmor-bzh v9 assembly (Table 4). Rlm1 was localised within an interval of approximately 4.94 Mbp containing 13 RGAs. Rlm3 and Rlm4 were placed within intervals of 16.79 Mbp (60 RGAs) and 3.71 Mbp (17 RGAs), respectively. Rlm7 and Rlm9 loci were localised within 16.02 Mbp (51 RGAs) and 5.35 Mbp (21 RGAs), respectively. The A02 (LepR1) and A10 (LepR2) RGAs were localised to regions 10.41 (7 RGAs) and 13.95 Mbp (29 RGAs), respectively. Rlm1 and Rlm4 were in the smallest region which covered 13 and 17 RGAs, respectively (Table 4).

Overall, we identified 100 RGAs within previously known regions for blackleg resistance of which 22 RGA were affected by CNV events. There were 64 RGAs overlapping Rlm1, Rlm3, Rlm4, Rlm7 and Rlm9 QTL on chromosome A07 of which 16 were affected by CNV events; 12 RLKs and 1 TNL were deleted, and 2 RLKs and 1 TNL were duplicated. On chromosome A02, out of 7 RLKs, two RLKs were deleted, on chromosome A10, out of 29 RGAs three RLKs were deleted, and one RLK was duplicated (Table 4).

## 4. Discussion

Recently, several studies have reported CNV events across various crop species, including rice [27,36], wheat [85], barley [86], maize [52,87], soybean [46], melon [88] and cannabis [89]. Most of these studies have linked CNV analysis with agronomic traits. Given that canola is a major crop and CNVs are among the major genomic structural variations and hotspots for genetic and phenotypic variation during environmental adaptation and population differentiation, we performed genome-wide analysis of CNV events of RGAs across eight canola cultivars. In total 563 RGAs overlapped with 1,137 CNV events of which the majority were deletions (704 deletions, 433 duplications). The higher number of deletions than duplications is consistent with other *B. napus* studies. Schiessl, Huettel, Kuehn, Reinhardt and Snowdon [29] have shown that deletions are more abundant than duplications in *B. napus* as genomes are known to reduce their gene space after polyploidisation [90].

Deletions abolish gene function, whereas duplications can cause an alteration in gene expression level [91] and thereby affect gene dosage. Kopec et al. (2021) showed in *B. napus* resistant and susceptible lines against clubroot that the transcript levels of the two TNL copies in the resistant line was twice the amount of the transcript level of one copy in the susceptible line, and this upregulation was most likely involved with the resistance response [55]. Therefore, duplications are more likely to change traits than point mutations or InDels [92].

We found more deletions in the C sub-genome than in the A sub-genome and more duplications in the A sub-genome than in the C sub-genome. These findings are consistent with earlier *B. napus* studies [29]. This might be due to the fact that the A sub-genome copies had been selected over the C sub-genome copies. For example, CNVs concerning copies of Bna.FLC, Bna.PHYA and Bna.GA3ox1 involve duplications in the A sub-genome and corresponding homoeologous deletions in the C sub-genome [93]. Another possible explanation for this genome bias might be due to the high transposon content and more active transposons in the C sub-genome [5,94]. Generally, due to high gene redundancy [29] and inter-sub-genomic homology [95], genomic rearrangements are common events in polyploid genomes. Our data suggest that CNVs larger than 1 kb but smaller than 5 kb are more frequent than other CNV sizes. Similar results were found in rice and maize where smaller CNVs (shorter than 10 kb) are more frequent than larger ones [36,96].

CNV numbers differ between species and between individuals of the same species. In this study, the chromosomes of all eight cultivars exhibited different numbers and patterns of CNV events. Similarly, Springer et al. (2009) identified more than 400 putative CNVs between Mo17 and B73 maize inbred lines distributed across all maize chromosomes [31]. Furthermore, Demeke and Eng (2018) investigated CNVs among three canola cultivars and found variability in gene copy numbers [97].

Although CNVs frequently overlap with protein-coding regions in plant genomes [95], little is known about the presence and phenotypic effects of CNVs in plants. Nevertheless, the nature of CNVs detected in maize suggests that they may have a significant impact on plant phenotypes, including disease response and heterosis [36]. We found that the majority of RGAs that were associated with CNV events are RLKs due to RLKs being the most abundant class of RGAs. RLKs and RLPs are primary components of the first line of plant immune response and mediate microbial elicitors pathogen/microbe-associated molecular pattern (PAMP/MAMP), triggered immunity (PTI/MTI) [98] to recognize broad spectra of pathogens [99]. In addition to defense mechanisms, RLKs and RLPs are also involved with developmental processes [98] including meristem and stomatal development [100,101] which can explain their abundance across the genomes.

It has been reported that the CNV of RGAs differ between species and within species [102,103], and this variability allows RGAs to recognize a wide range of effector proteins [104]. Therefore, a high copy number of RGAs should be beneficial to guard against the genetic diversity of pathogens.

We found that genes localized in physical clusters exhibit more CNV than singletons, which is consistent with a previous study in soybean [105]. RGAs in plants tend to be physically clustered in genomes [106]. For example, approximately 66% of resistance genes in Arabidopsis [107] and 76% in rice [108] were found in physical clusters. In addition, Yr genes responsible for resistance against wheat yellow rust were found to be physically clustered [109]. Similar to our findings, it has been previously reported that the majority of RGAs within a cluster belong to the same subfamily [110,111] and can have different rates and patterns of variation [112]. Genes in physical clusters may have adaptive advantages derived from rapid evolution due to rearrangement [52]. The results revealed that CNVs are distributed throughout the genome and CNV affected genes were more likely to be found in physical clusters. Thus, gene clustering may be a critical feature of the generation of novel resistance specificities through gene deletion or duplication.

Several regions that carry blackleg resistance genes have been identified in *B. napus* cultivars [80,83,113,114]. We identified 22 RGAs within the regions associated with blackleg resistance affected by CNV events, potentially leading to different levels of disease resistance in cultivars. Identification of RGA candidates and their structural variation will assist with RGA mapping and a better understanding of RGA evolution and functionality which is beneficial for genes identification and their application breeding programs.

To conclude, whole-genome sequencing was used to investigate CNV events of RGAs across eight blackleg resistant *B. napus* cultivars. The outcomes reveal that CNV events are a key type of genomic variation that may play an important role in disease resistance. The results constitute a valuable genome-wide variation resource of *B. napus* for future research on phenotypic variation and breeding. The results also provide insights into the evolution, formation and distribution of resistance genes in *B. napus*.

## Figures and Tables

**Table 1:** The number and percentage of CNV events in RGAs (i.e., deletion, duplication and both deletion and duplication) in eight *B. napus* cultivars.

Cultivars	Deletion	Duplication	Total	Both
Ascona	48 (56.47%)	37 (43.53%)	85	11 (12.94%)
English Giant	87 (55.77%)	69 (44.23%)	156	29 (18.59%)
Hansen × Gaspard	33 (52.38%)	30 (47.62%)	63	17 (26.98%)
Milena	67 (63.81%)	38 (36.19%)	105	21 (20.00%)
Pacific	58 (50%)	58 (50%)	116	19 (16.38%)
Pirola	77 (65.25%)	41 (34.75%)	118	14 (11.86%)
Tina	163 (68.20%)	76 (31.80%)	239	34 (14.23%)
Wilhelmsburger	171 (67.06%)	84 (32.94%)	255	43 (16.86%)
Total	704 (61.92%)	433 (38.08%)	1137	188 (16.53%)

**Table 2:** The number of RGAs with the same CNV type in eight *B. napus* cultivars.

	Ascona	English Giant	Hansen × Gaspard	Milena	Pacific	Pirola	Tina	Wilhelmsburger
Ascona	-							
English Giant	24	-						
Hansen × Gaspard	17	11	-					
Milena	30	36	27	-				
Pacific	36	25	22	42	-			
Pirola	37	28	23	42	47	-		
Tina	31	52	24	42	47	38	-	
Wilhelmsburger	37	65	26	51	43	54	126	-

**Table 3:** The number and percentage of RGAs with CNV shared among the cultivars.

Shared between	1 Cultivar	2 Cultivars	3 Cultivars	4 Cultivars	5 Cultivars	6 Cultivars	7 Cultivars	8 Cultivars	Total
RGAs	262	157	81	27	15	14	5	2	563
	46.54%	27.89%	14.39%	4.80%	2.66%	2.49%	0.89%	0.36%	100%

**Table 4:** RGA candidates and RGA affected by CNV events underlying reported regions for blackleg resistance genes.

Locus	Marker	Reference	Chromosome	Start (Mbp)	End (Mbp)	Length (Mbp)	RGA Candidates	RGA Affected by CNV
*Rlm1*	Na12A02	-		22.35	27.29	4.94	TN 1, OTHER 1, TX 1, RLK 8, NL 1, CNL 1	RLK 1 (1 del)
	Ol12-E03A	Delourme et al., 2004 [79]						
	CB10544A	Raman, Taylor, Lindbeck et al., 2012 [80]	A7					
	Ra2-A05b	-						
	BSR + KASP							
*Rlm3*	BnGMS147b	Delourme et al., 2004 [79]	A7	8.97	25.75	16.79	RLK 44, RLP 5, NL 4, CNL 1, TNL 3, TN 1, OTHER 1, TX 1	RLK 14 (12 del, 2 dup), TNL 2 (1 del, 1 dup)
	IGF0504f_F	Leflon et al., 2007 [82]						
*Rlm4*	BRMS040	Raman, Taylor, Marcroft et al., 2012) [80]	A7	11.49	15.20	3.71	RLK 15, RLP 2	RLK 9 (8 del, 1 dup)
	Na12-E11b							
*Rlm7*	sR7018	Larkan et al., 2016 [83]	A7	12.16	28.19	16.02	RLK 34, RLP 5, NL 4, CNL 2, TNL 3, TN 1, OTHER 1, TX 1	RLK 8 (7 del, 1 dup), TNL 2 (1 del, 1dup)
*Rlm9*	BnGMS665	Delourme et al., 2004 [79]	A7	13.76	19.11	5.35	RLK 16, RLP 3, NL 2	RLK 4 (4 del)
	BnGMS147b							
*LepR1*	FlankingMarkers	Larkan et al., 2016 [83]	A02	10.02	20.43	10.41	RLK 7	RLK 2 (2 del)
*LepR2*	FlankingMarkers	Larkan et al., 2016 [83]	A10	0.20	14.15	13.95	RLK 17, RLP 6, TX 1, NL 1, CN 2, CNL 1, NBS 1	RLK 4 (3 del, 1dup)

## Data Availability

Not applicable.

## Supplementary Materials

The following supporting information can be found at https://www.mdpi.com/article/10.3390/genes13112037/s1. Figure S1: The position of CNV events (red and blue lines represents deletions and duplications, respectively) across the chromosomes of eight *B. napus* cultivars. The tracks from outer to inner show chromosomes, Ascona, English Giant, Hansen × Gaspard, Hansen × Gaspard, Milena, Pa Pacific, Pirola, Tina and Wilhelmsburger; Table S1: Chromosomal distribution of CNV events in eight *B. napus* cultivars; Table S2: Characteristics of CNVs including CNV number, deletion to duplication ratio, average CNV size and percentage of CNVs larger or smaller than average in eight *B. napus* cultivars; Table S3: The number of RGAs affected by CNV events in eight *B. napus* lines; Table S4: The number of singletons and clustered RGAs affected by CNV across 563 RGAs; Table S5: Distribution and number of the singletons and clustered RGAs affected by CNV across the chromosomes.

## References

[1] Golicz A.A., Bayer P.E., Bhalla P.L., Batley J., Edwards D. (2020). Pangenomics comes of age: From bacteria to plant and animal applications. Trends Genet..

[2] Bayer P.E., Golicz A.A., Scheben A., Batley J., Edwards D. (2020). Plant pan-genomes are the new reference. Nat. Plants.

[3] Dolatabadian A., Bayer P.E., Tirnaz S., Hurgobin B., Edwards D., Batley J. (2020). Characterization of disease resistance genes in the Brassica napus pangenome reveals significant structural variation. Plant Biotechnol. J..

[4] Hurgobin B., Golicz A.A., Bayer P.E., Chan C.K., Tirnaz S., Dolatabadian A., Schiessl S.V., Samans B., Montenegro J.D., Parkin I.A.P. (2018). Homoeologous exchange is a major cause of gene presence/absence variation in the amphidiploid Brassica napus. Plant Biotechnol. J..

[5] Bayer P.E., Scheben A., Golicz A.A., Yuan Y., Faure S., Lee H., Chawla H.S., Anderson R., Bancroft I., Raman H. (2021). Modelling of gene loss propensity in the pangenomes of three Brassica species suggests different mechanisms between polyploids and diploids. Plant Biotechnol. J..

[6] Golicz A.A., Bayer P.E., Barker G.C., Edger P.P., Kim H., Martinez P.A., Chan C.K.K., Severn-Ellis A., McCombie W.R., Parkin I.A.P. (2016). The pangenome of an agronomically important crop plant Brassica oleracea. Nat. Commun..

[7] Bayer P.E., Golicz A.A., Tirnaz S., Chan C.K., Edwards D., Batley J. (2019). Variation in abundance of predicted resistance genes in the *Brassica oleracea* pangenome. Plant Biotechnol. J..

[8] Montenegro J.D., Golicz A.A., Bayer P.E., Hurgobin B., Lee H., Chan C.K.K., Visendi P., Lai K., Doležel J., Batley J. (2017). The pangenome of hexaploid bread wheat. Plant J..

[9] Hu H., Scheben A., Verpaalen B., Tirnaz S., Bayer P.E., Hodel R.G.J., Batley J., Soltis D.E., Soltis P.S., Edwards D. (2021). Amborella gene presence/absence variation is associated with abiotic stress responses that may contribute to environmental adaptation. New Phytol..

[10] Zhao J., Bayer P., Ruperao P., Saxena R., Khan A., Golicz A., Nguyen H., Batley J., Edwards D., Varshney R. (2020). Trait associations in the pangenome of pigeon pea (*Cajanus cajan*). Plant Biotechnol. J..

[11] Yu J., Golicz A.A., Lu K., Dossa K., Zhang Y., Chen J., Wang L., You J., Fan D., Edwards D. (2019). Insight into the evolution and functional characteristics of the pan-genome assembly from sesame landraces and modern cultivars. Plant Biotechnol. J..

[12] Zhao Q., Feng Q., Lu H., Li Y., Wang A., Tian Q., Zhan Q., Lu Y., Zhang L., Huang T. (2018). Pan-genome analysis highlights the extent of genomic variation in cultivated and wild rice. Nat. Genet..

[13] Zhou Y., Chebotarov D., Kudrna D., Llaca V., Lee S., Rajasekar S., Mohammed N., Al-Bader N., Sobel-Sorenson C., Parakkal P. (2020). A platinum standard pan-genome resource that represents the population structure of Asian rice. Sci. Data.

[14] Liu Y., Du H., Li P., Shen Y., Peng H., Liu S., Zhou G.A., Zhang H., Liu Z., Shi M. (2020). Pan-Genome of Wild and Cultivated Soybeans. Cell.

[15] Bayer P.E., Valliyodan B., Hu H., Marsh J.I., Yuan Y., Vuong T.D., Patil G., Song Q., Batley J., Varshney R.K. (2021). Sequencing the USDA core soybean collection reveals gene loss during domestication and breeding. Plant Genome.

[16] Torkamaneh D., Lemay M.A., Belzile F. (2021). The pan-genome of the cultivated soybean (PanSoy) reveals an extraordinarily conserved gene content. Plant Biotechnol. J..

[17] Rijzaani H., Bayer P.E., Rouard M., Doležel J., Batley J., Edwards D. (2021). The pangenome of banana highlights differences between genera and genomes. Plant Genome.

[18] Varshney R., Nayak S., May G., Jackson S. (2009). Next-generation sequencing technologies and their implications for crop genetics and breeding. Trends Biotechnol..

[19] Huang X., Lu T., Han B. (2013). Resequencing rice genomes: An emerging new era of rice genomics. Trends Genet..

[20] Wang H., Chai Z., Hu D., Ji Q., Xin J., Zhang C., Zhong J. (2019). A global analysis of CNVs in diverse yak populations using whole-genome resequencing. BMC Genom..

[21] Murthy M., Veerappa A.M., Seshachalam K., Ramachandra N. (2016). High-resolution arrays reveal burden of copy number variations on Parkinson disease genes associated with increased disease risk in random cohorts. Neurol. Res..

[22] Gamazon E., Stranger B. (2015). The impact of human copy number variation on gene expression. Brief. Funct. Genom..

[23] Hastings P., Lupski J., Rosenberg S., Ira G. (2009). Mechanisms of change in gene copy number. Nat. Rev. Genet..

[24] Schrider D., Hahn M. (2010). Gene copy-number polymorphism in nature. Proc. R. Soc. B Biol. Sci..

[25] Yan Y., Yang N., Cheng H., Song J., Qu L. (2015). Genome-wide identification of copy number variations between two chicken lines that differ in genetic resistance to Marekâ€™s disease. BMC Genom..

[26] Hull R., Cruz C., Jack C., Houseley J. (2017). Environmental change drives accelerated adaptation through stimulated copy number variation. PLoS Biol..

[27] Bai Z., Chen J., Liao Y., Wang M., Liu R., Ge S., Wing R., Chen M. (2016). The impact and origin of copy number variations in the Oryza species. BMC Genom..

[28] DeBolt S. (2010). Copy number variation shapes genome diversity in Arabidopsis over immediate family generational scales. Genome Biol. Evol..

[29] Schiessl S., Huettel B., Kuehn D., Reinhardt R., Snowdon R.J. (2017). Targeted deep sequencing of flowering regulators in Brassica napus reveals extensive copy number variation. Sci. Data.

[30] Redon R., Ishikawa S., Fitch K., Feuk L., Perry G., Andrews T., Fiegler H., Shapero M., Carson A., Chen W. (2006). Global variation in copy number in the human genome. Nature.

[31] Springer N., Ying K., Fu Y., Ji T., Yeh C., Jia Y., Wu W., Richmond T., Kitzman J., Rosenbaum H. (2009). Maize inbreds exhibit high levels of copy number variation. PLoS Genet..

[32] Swanson-Wagner R., Eichten S., Kumari S., Tiffin P., Stein J., Ware D., Springer N. (2010). Pervasive gene content variation and copy number variation in maize and its undomesticated progenitor. Genome Res..

[33] Maron L., Guimaraes C., Kirst M., Albert P., Birchler J., Bradbury P., Buckler E., Coluccio A., Danilova T., Kudrna D. (2013). Aluminum tolerance in maize is associated with higher MATE1 gene copy number. Proc. Natl. Acad. Sci. USA.

[34] Zmienko A., Samelak-Czajka A., Kozlowski P., Szymanska M., Figlerowicz M. (2016). Arabidopsis thaliana population analysis reveals high plasticity of the genomic region spanning MSH2, AT3G18530 and AT3G18535 genes and provides evidence for NAHR-driven recurrent CNV events occurring in this location. BMC Genom..

[35] Zmienko A., Marszalek-Zenczak M., Wojciechowski P., Samelak-Czajka A., Luczak M., Kozlowski P., Karlowski W.M., Figlerowicz M. (2020). AthCNV: A Map of DNA Copy Number Variations in the Arabidopsis Genome [OPEN]. Plant Cell.

[36] Yu P., Wang C., Xu Q., Feng Y., Yuan X., Yu H., Wang Y., Tang S., Wei X. (2011). Detection of copy number variations in rice using array-based comparative genomic hybridization. BMC Genom..

[37] Zhao F., Wang Y., Zheng J., Wen Y., Qu M., Kang S., Wu S., Deng X., Hong K., Li S. (2020). A genome-wide survey of copy number variations reveals an asymmetric evolution of duplicated genes in rice. BMC Biol..

[38] Saintenac C., Jiang D., Akhunov E. (2011). Targeted analysis of nucleotide and copy number variation by exon capture in allotetraploid wheat genome. Genome Biol..

[39] Diaz A., Zikhali M., Turner A., Isaac P., Laurie D. (2012). Copy number variation affecting the photoperiod-B1 and vernalization-A1 genes is associated with altered flowering time in wheat. PLoS ONE.

[40] Zhang Q., Saghai Maroof M.A., Allard R. (1990). Effects on adaptedness of variations in ribosomal DNA copy number in populations of wild barley. Proc. Natl. Acad. Sci. USA.

[41] Sutton T., Baumann U., Hayes J., Collins N., Shi B., Schnurbusch T., Hay A., Mayo G., Pallotta M., Tester M. (2007). Boron-toxicity tolerance in barley arising from efflux transporter amplification. Science.

[42] Nitcher R., Distelfeld A., Tan C., Yan L., Dubcovsky J. (2013). Increased copy number at the HvFT1 locus is associated with accelerated flowering time in barley. Mol. Genet. Genom..

[43] Datta S., Jankowicz-Cieslak J., Nielen S., Ingelbrecht I., Till B. (2018). Induction and recovery of copy number variation in banana through gamma irradiation and low-coverage whole-genome sequencing. Plant Biotechnol. J..

[44] Alonge M., Wang X., Benoit M., Soyk S., Pereira L., Zhang L., Suresh H., Ramakrishnan S., Maumus F., Ciren D. (2020). Major Impacts of widespread structural variation on gene expression and crop improvement in tomato. Cell.

[45] Cook D., Lee T., Guo X., Melito S., Wang K., Bayless A., Wang J., Hughes T., Willis D., Clemente T. (2012). Copy number variation of multiple genes at Rhg1 mediates nematode resistance in soybean. Science.

[46] Lee T., Diers B., Hudson M. (2016). An efficient method for measuring copy number variation applied to improvement of nematode resistance in soybean. Plant J..

[47] Bakker E., Toomajian C., Kreitman M., Bergelson J. (2006). A genome-wide survey of R gene polymorphisms in Arabidopsis. Plant Cell.

[48] Shen J., Araki H., Chen L., Chen J., Tian D. (2006). Unique evolutionary mechanism in R genes under the presence/absence polymorphism in Arabidopsis thaliana. Genetics.

[49] Xu X., Liu X., Ge S., Jensen J., Hu F., Li X., Dong Y., Gutenkunst R., Fang L., Huang L. (2012). Resequencing 50 accessions of cultivated and wild rice yields markers for identifying agronomically important genes. Nat. Biotechnol..

[50] González V., Aventin N., Centeno E., Puigdomenech P. (2013). High presence/absence gene variability in defence-related gene clusters of Cucumis melo. BMC Genom..

[51] Lin X., Zhang Y., Kuang H., Chen J. (2013). Frequent loss of lineages and deficient duplications accounted for low copy number of disease resistance genes in Cucurbitaceae. BMC Genom..

[52] Hu Y., Ren J., Peng Z., Umana A., Le H., Danilova T., Fu J., Wang H., Robertson A., Hulbert S. (2018). Analysis of extreme phenotype bulk copy number variation. Front. Plant Sci..

[53] Lee O., Kumar I., Diers B., Hudson M. (2015). Evolution and selection of Rhg1, a copy-number variant nematode-resistance locus. Mol. Ecol..

[54] Gabur I., Chawla H.S., Lopisso D.T., von Tiedemann A., Snowdon R.J., Obermeier C. (2020). Gene presence-absence variation associates with quantitative *Verticillium longisporum* disease resistance in *Brassica napus*. Sci. Rep..

[55] Kopec P.M., Mikolajczyk K., Jajor E., Perek A., Nowakowska J., Obermeier C., Chawla H.S., Korbas M., Bartkowiak-Broda I., Karlowski W.M. (2021). Local Duplication of TIR-NBS-LRR Gene Marks Clubroot Resistance in Brassica napus cv. Tosca. Front. Plant Sci..

[56] Chalhoub B., Denoeud F., Liu S., Parkin I., Tang H., Wang X., Chiquet J., Belcram H., Tong C., Samans B. (2014). Early allopolyploid evolution in the post-Neolithic Brassica napus oilseed genome. Science.

[57] Bhattarai K., Wang W., Cao Z., Deng Z. (2018). Comparative Analysis of Impatiens Leaf Transcriptomes Reveal Candidate Genes for Resistance to Downy Mildew Caused by Plasmopara obducens. Int. J. Mol. Sci..

[58] Marti F., Saski C., Manganaris G., Gasic K., Crisosto C. (2018). Genomic sequencing of Japanese plum. Front. Plant Sci..

[59] Mason A., Snowdon R. (2016). Oilseed rape: Learning about ancient and recent polyploid evolution from a recent crop species. Plant Biol..

[60] Szadkowski E., Eber F., Huteau V., Lode M., Coriton O., Jenczewski E., Chevre A. (2011). Polyploid formation pathways have an impact on genetic rearrangements in resynthesized Brassica napus. New Phytol..

[61] Nicolas S., Monod H., Eber F., Chevre A., Jenczewski E. (2012). Non-random distribution of extensive chromosome rearrangements in Brassica napus depends on genome organization. Plant J..

[62] Jones J.D.G., Dangl J.L. (2006). The plant immune system. Nature.

[63] Ecke W., Clemens R., Honsdorf N., Becker H.C. (2010). Extent and structure of linkage disequilibrium in canola quality winter rapeseed (*Brassica napus* L.). Theor. Appl. Genet. Theor. Und Angew. Genet..

[64] Ganya S., Svotwa E., Katsaruware R.D. (2018). Performance of two Rape (*Brassica napus*) cultivars under different fertilizer management levels in the smallholder sector of Zimbabwe. Int. J. Agron..

[65] Lammerink J., Hart R. (1985). ‘Tina’, a new swede cultivar with resistance to dry rot and clubroot. N. Z. J. Exp. Agric..

[66] Hasan M.J., Rahman H. (2016). Genetics and molecular mapping of resistance to *Plasmodiophora brassicae* pathotypes 2, 3, 5, 6, and 8 in rutabaga *(Brassica napus* var. napobrassica). Genome.

[67] Wilch A. (2018). Characterisation of Genotypic and Tissue Specific Resistance in Oilseed Rape (B. napus) Against Sclerotinia sclerotiorum.

[68] Lammerink J. (1965). Six pathogenic races of Plasmodiophora brassicae Wor. in New Zealand. N. Z. J. Agric. Res..

[69] Knights B.A. (1968). Studies in the cruciferae: Sterols in pollen of *Brassica napus* L.. Phytochemistry.

[70] Stonard J.F., Downes K., Pirie E., Fitt B.D.L., Evans N. Development of phoma stem canker (*Leptosphaeria maculans*) and light leaf spot (*Pyrenopeziza brassicae*) on current and historical oilseed rape cultivars in 2003/04, 2004/05 and 2005/06 UK growing seasons. Proceedings of the 12th International Rapeseed Congress.

[71] Bolger A., Lohse M., Usadel B. (2014). Trimmomatic: A flexible trimmer for Illumina sequence data. Bioinformatics.

[72] Chiang C., Layer R., Faust G., Lindberg M., Rose D., Garrison E., Marth G., Quinlan A.R., Hall I.M. (2015). SpeedSeq: Ultra-fast personal genome analysis and interpretation. Nat. Methods.

[73] Li H., Durbin R. (2009). Fast and accurate short read alignment with Burrows-Wheeler transform. Bioinformatics.

[74] Tarasov A., Vilella A., Cuppen E., Nijman I., Prins P. (2015). Sambamba: Fast processing of NGS alignment formats. Bioinformatics.

[75] Abyzov A., Urban A., Snyder M., Gerstein M. (2011). CNVnator: An approach to discover, genotype, and characterize typical and atypical CNVs from family and population genome sequencing. Genome Res..

[76] Li H. (2011). A statistical framework for SNP calling, mutation discovery, association mapping and population genetical parameter estimation from sequencing data. Bioinformatics.

[77] Quinlan A., Hall I. (2010). BEDTools: A flexible suite of utilities for comparing genomic features. Bioinformatics.

[78] Li P., Quan X., Jia G., Xiao J., Cloutier S., You F. (2016). RGAugury: A pipeline for genome-wide prediction of resistance gene analogs. BMC Genom..

[79] Delourme R., Pilet-Nayel M., Archipiano M., Horvais R., Tanguy X., Rouxel T., Brun H., Renard M., Balesdent M. (2004). A Cluster of Major Specific Resistance Genes to Leptosphaeria maculans in Brassica napus. Phytopathology.

[80] Raman R., Taylor B., Marcroft S., Stiller J., Eckermann P., Coombes N., Rehman A., Lindbeck K., Luckett D., Wratten N. (2012). Molecular mapping of qualitative and quantitative loci for resistance to Leptosphaeria maculans causing blackleg disease in canola. Crop Pasture Sci..

[81] Raman R., Taylor B., Lindbeck K., Coombes N., Barbulescu D., Salisbury P., Raman H. (2012). Molecular mapping and validation of Rlm1 gene for resistance to Leptosphaeria maculans in canola. Crop Pasture Sci..

[82] Leflon M., Brun H., Eber F., Delourme R., Lucas M., Vallée P., Ermel M., Balesdent M., Chèvre A. (2007). Detection, introgression and localization of genes conferring specific resistance to Leptosphaeria maculans from Brassica rapa into B. TAG Theor. Appl. Genet. Theor. Und Angew. Genet..

[83] Larkan N., Raman H., Lydiate D., Robinson S., Yu F., Barbulescu D., Raman R., Luckett D., Burton W., Wratten N. (2016). Multi-environment QTL studies suggest a role for cysteine-rich protein kinase genes in quantitative resistance to blackleg disease in Brassica napus. BMC Plant Biol..

[84] Camacho C., Coulouris G., Avagyan V., Ma N., Papadopoulos J., Bealer K., Madden T.L. (2009). BLAST+: Architecture and applications. BMC Bioinform..

[85] Wurschum T., Longin C., Hahn V., Tucker M., Leiser W. (2017). Copy number variations of CBF genes at the Fr-A2 locus are essential components of winter hardiness in wheat. Plant J..

[86] Francia E., Morcia C., Pasquariello M., Mazzamurro V., Milc J., Rizza F., Terzi V., Pecchioni N. (2016). Copy number variation at the HvCBF4-HvCBF2 genomic segment is a major component of frost resistance in barley. Plant Mol. Biol..

[87] Dong J., Feng Y., Kumar D., Zhang W., Zhu T., Luo M., Messing J. (2016). Analysis of tandem gene copies in maize chromosomal regions reconstructed from long sequence reads. Proc. Natl. Acad. Sci. USA.

[88] Demirci S., Fuentes R.R., Dooijeweert W.V., Aflitos S., Schijlen E., Hesselink T., de Ridder D., Dijk A.D.J.V., Peters S. (2021). Chasing breeding footprints through structural variations in Cucumis melo and wild relatives. G3 Genes|Genomes|Genet..

[89] McKernan K.J., Helbert Y., Kane L.T., Ebling H., Zhang L., Liu B., Eaton Z., McLaughlin S., Kingan S., Baybayan P. (2020). Sequence and annotation of 42 cannabis genomes reveals extensive copy number variation in cannabinoid synthesis and pathogen resistance genes. bioRxiv.

[90] Sankoff D., Zheng C., Zhu Q. (2010). The collapse of gene complement following whole genome duplication. BMC Genom..

[91] Lovene M., Zhang T., Lou Q., Buell C., Jiang J. (2013). Copy number variation in potato-an asexually propagated autotetraploid species. Plant J..

[92] Schiessl S., Katche E., Ihien E., Chawla H., Mason A. (2018). The role of genomic structural variation in the genetic improvement of polyploid crops. Crop J..

[93] Schiessl S., Huettel B., Kuehn D., Reinhardt R., Snowdon R. (2017). Post-polyploidisation morphotype diversification associates with gene copy number variation. Sci. Rep..

[94] Parkin I., Koh C., Tang H., Robinson S., Kagale S., Clarke W., Town C., Nixon J., Krishnakumar V., Bidwell S. (2014). Transcriptome and methylome profiling reveals relics of genome dominance in the mesopolyploid Brassica oleracea. Genome Biol..

[95] Zmienko A., Samelak A., Kozlowski P., Figlerowicz M. (2014). Copy number polymorphism in plant genomes. Theor. Appl. Genet..

[96] Beló A., Beatty M., Hondred D., Fengler K., Li B., Rafalski A. (2010). Allelic genome structural variations in maize detected by array comparative genome hybridization. Theor. Appl. Genet..

[97] Demeke T., Eng M. (2018). Effect of endogenous reference genes on digital PCR assessment of genetically engineered canola events. Biomol. Detect. Quantif..

[98] Sekhwal M., Li P., Lam I., Wang X., Cloutier S., You F. (2015). Disease resistance gene analogs. Int. J. Mol. Sci..

[99] Chisholm S., Coaker G., Day B., Staskawicz B. (2006). Host-microbe interactions: Shaping the evolution of the plant immune response. Cell.

[100] Jeong S., Trotochaud A.E., Clark S.E. (1999). The Arabidopsis CLAVATA2 gene encodes a receptor-like protein required for the stability of the CLAVATA1 receptor-like kinase. Plant Cell.

[101] Nadeau J.A., Sack F.D. (2002). Control of stomatal distribution on the Arabidopsis leaf surface. Science.

[102] Yang S., Feng Z., Zhang X., Jiang K., Jin X., Hang Y., Chen J., Tian D. (2006). Genome-wide investigation on the genetic variations of rice disease resistance genes. Plant Mol. Biol..

[103] Zhang M., Wu Y., Lee M., Liu Y., Rong Y., Santos T., Wu C., Xie F., Nelson R., Zhang H. (2010). Numbers of genes in the NBS and RLK families vary by more than four-fold within a plant species and are regulated by multiple factors. Nucleic Acids Res..

[104] Kim J., Lim C.J., Lee B., Choi J., Oh S., Ahmad R., Kwon S., Ahn J., Hur C. (2012). A Genome-wide comparison of NB-LRR type of resistance gene analogs. Mol. Cells.

[105] McHale L., Haun W., Xu W., Bhaskar P., Anderson J., Hyten D., Gerhardt D., Jeddeloh J., Stupar R. (2012). Structural variants in the soybean genome localize to clusters of biotic stress response genes. Plant Physiol..

[106] McHale L., Tan X., Koehl P., Michelmore R. (2006). Plant NBS-LRR proteins: Adaptable guards. Genome Biol..

[107] Meyers B., Kozik A., Griego A., Kuang H., Michelmore R. (2003). Genome-wide analysis of NBS-LRR-encoding genes in Arabidopsis. Plant Cell.

[108] Luo S., Zhang Y., Hu Q., Chen J., Li K., Lu C., Liu H., Wang W., Kuang H. (2012). Dynamic nucleotide-binding site and leucine-rich repeat-encoding genes in the grass family. Plant Physiol..

[109] Marchal C., Zhang J., Zhang P., Fenwick P., Steuernagel B., Adamski N., Boyd L., McIntosh R., Wulff B., Berry S. (2018). BED-domain containing immune receptors confer diverse resistance spectra to yellow rust. Nat. Plants.

[110] Ashfield T., Egan A., Pfeil B., Chen N., Podicheti R., Ratnaparkhe M., Ameline-Torregrosa C., Denny R., Cannon S., Doyle J. (2012). Evolution of a complex disease resistance gene cluster in diploid Phaseolus and tetraploid Glycine. Plant Physiol..

[111] Alamery S., Tirnaz S., Bayer P., Tollenaere R., Chalhoub B., Edwards D., Batley J. (2017). Genome-wide identification and comparative analysis of NBS-LRR resistance genes in Brassica napus. Crop Pasture Sci..

[112] Kuang H., Woo S., Meyers B., Nevo E., Michelmore R. (2004). Multiple genetic processes result in heterogeneous rates of evolution within the major cluster disease resistance genes in lettuce. Plant Cell.

[113] Jestin C., Lodé M., Vallée P., Domin C., Falentin C., Horvais R., Coedel S., Manzanares-Dauleux M., Delourme R. (2011). Association mapping of quantitative resistance for Leptosphaeria maculans in oilseed rape. Mol. Breed..

[114] Huang Y., Jestin C., Welham S., King G., Manzanares-Dauleux M., Fitt B., Delourme R. (2016). Identification of environmentally stable QTL for resistance against Leptosphaeria maculans in oilseed rape. TAG Theor. Appl. Genet. Theor. Und Angew. Genet..

