# Supplementary material for: Copy Number Variation among Resistance Genes Analogues in Brassica napus
Source: Genes (Basel). 2022 Nov 4;13(11):2037. doi: 10.3390/genes13112037 (PMC9690292; doi:10.3390/genes13112037)
Supplement: Supplementary file 1 [file genes-13-02037-s001.zip › genes-1983596-supplementary.pdf]

Table S1: Chromosomal distribution of CNV events in eight *B. napus* lines.

Lines	Ascona		English Giant		Hansen × Gaspard		Milena		Pacific		Pirola		Tina		Wilhelsburger				
Chromosomes	Del	Dup	Del	Dup	Del	Dup	Del	Dup	Del	Dup	Del	Dup	Del	Dup	Del	Dup	**Sum Del**	**Sum Dup**	**Total**
A01	0	0	3	2	2	3	2	5	2	2	1	1	5	3	6	7	**21**	**23**	**44**
A02	3	4	3	1	2	0	9	0	3	2	4	3	10	8	12	0	**46**	**18**	**64**
A03	0	1	6	0	2	4	1	3	2	3	2	12	2	5	4	12	**19**	**40**	**59**
A04	1	1	2	1	1	1	1	1	1	3	1	1	4	2	2	0	**13**	**10**	**23**
A05	0	1	3	1	1	1	1	0	0	0	2	0	9	0	5	1	**21**	**4**	**25**
A06	3	4	9	25	1	2	6	3	2	3	7	1	3	5	3	4	**34**	**47**	**81**
A07	4	1	8	1	1	0	3	1	3	1	2	0	7	1	5	0	**33**	**5**	**38**
A08	4	3	5	1	2	3	8	3	9	3	7	2	8	2	7	2	**50**	**19**	**69**
A09	3	0	8	17	10	1	6	1	5	16	7	2	15	24	19	21	**73**	**82**	**155**
A10	2	0	0	0	0	0	1	0	1	1	1	0	0	1	2	1	**7**	**3**	**10**
**Total**	**20**	**15**	**47**	**49**	**22**	**15**	**38**	**17**	**28**	**34**	**34**	**22**	**63**	**51**	**65**	**48**	**317**	**251**	**568**
C01	7	2	5	0	1	0	4	0	0	3	2	0	7	0	10	0	**36**	**5**	**41**
C02	2	1	11	5	0	1	6	2	5	3	4	2	18	7	7	11	**53**	**32**	**85**
C03	4	7	6	0	6	3	3	2	2	5	17	6	8	7	20	3	**66**	**33**	**99**
C04	1	2	1	1	0	2	2	4	2	3	2	1	1	4	9	2	**18**	**19**	**37**
C05	0	3	1	5	1	1	0	3	0	3	2	2	5	2	2	6	**11**	**25**	**36**
C06	11	1	0	0	2	1	1	2	11	2	11	3	11	1	18	0	**65**	**10**	**75**
C07	2	1	3	0	0	0	2	2	0	2	1	1	3	2	2	2	**13**	**10**	**23**
C08	0	4	0	1	0	2	4	3	6	0	2	1	30	1	16	0	**58**	**12**	**70**
C09	1	1	13	8	1	5	7	3	4	3	2	3	17	1	22	12	**67**	**36**	**103**
**Total**	**28**	**22**	**40**	**20**	**11**	**15**	**29**	**21**	**30**	**24**	**43**	**19**	**100**	**25**	**106**	**36**	**387**	**182**	**569**
**Grand total**	**48**	**37**	**87**	**69**	**33**	**30**	**67**	**38**	**58**	**58**	**77**	**41**	**163**	**76**	**171**	**84**	**704**	**433**	**1137**

Table S2: Characteristics of CNVs including CNV number, deletion to duplication ratio, average CNV size and percentage of CNVs larger or smaller than average in eight *B. napus* lines.

	Ascona		English Giant		HANSEN x GASPARD		Milena	
	Del	Dup	Del	Dup	Del	Dup	Del	Dup
CNV number	48	37	87	69	33	30	67	38
Deletion to duplication ratio	1.29	-	1.26	-	1.1	-	1.76	-
Average CNV size (kb)	3.06	1.91	3.54	3.37	2.35	3.15	4.90	1.93
Median CNV size (kb)	2.10	1.75	2.30	2.92	1.62	2.82	2.81	1.94
Larger than average (%)	37.5	43.24	24.14	28.99	39.39	36.67	23.88	50
Smaller than average (%)	62.5	56.76	75.86	71.01	60.61	63.33	76.12	50
	Pacific		Pirola		Tina		Wilhelsburger	
	Del	Dup	Del	Dup	Del	Dup	Del	Dup
CVN number	58	58	77	41	163	76	171	84
Deletion to duplication ratio	1	-	1.87	-	2.14	-	2.03	-
Average CNV size (kb)	2.73	2.69	3.44	2.72	3.73	2.62	4.03	2.56
Median CNV size (kb)	1.99	2.19	1.92	2.19	2.32	2.59	2.41	2.52
Larger than average (%)	37.93	39.66	28.57	39.02	25.15	50	22.81	50
Smaller than average (%)	62.07	60.34	71.43	60.98	74.85	50	77.19	50

Table S3: The number of RGAs affected by CNV events in eight *B. napus* lines.

	Ascona		English Giant		Hansen × Gaspard		Milena		Pacific		Pirola		Tina		Wilhelsburger				
RGAs	Del	Dup	Del	Dup	Del	Dup	Del	Dup	Del	Dup	Del	Dup	Del	Dup	Del	Dup	**Sum of Del**	**Sum of Dup**	**Total**
CN	1	2	5	2	0	0	2	1	1	1	3	0	2	3	7	2	**21**	**11**	**32**
CNL	5	2	2	3	0	2	5	4	2	3	7	1	10	7	10	6	**41**	**28**	**69**
NBS	1	0	5	0	1	1	5	1	2	2	3	1	7	1	6	5	**30**	**11**	**41**
NL	6	1	8	2	3	0	8	3	7	7	11	1	12	4	11	5	**66**	**23**	**89**
OTHER	0	0	1	1	1	0	1	0	1	0	2	0	1	1	4	0	**11**	**2**	**13**
TN	4	0	2	0	2	0	0	0	2	0	3	0	5	1	5	0	**23**	**1**	**24**
TNL	1	3	0	1	0	5	7	3	6	4	4	6	16	4	12	0	**46**	**26**	**72**
TX	2	0	1	1	2	0	3	0	2	0	2	1	9	1	9	2	**30**	**5**	**35**
**Sum**	**20**	**8**	**24**	**10**	**9**	**8**	**31**	**12**	**23**	**17**	**35**	**10**	**62**	**22**	**64**	**20**	**268**	**107**	**375**
RLK	20	23	48	50	16	18	24	17	27	31	32	23	73	41	79	51	**319**	**254**	**573**
RLP	8	6	15	9	8	4	12	9	8	10	10	8	28	13	28	13	**117**	**72**	**189**
**Sum**	**28**	**29**	**63**	**59**	**24**	**22**	**36**	**26**	**35**	**41**	**42**	**31**	**101**	**54**	**107**	**64**	**436**	**326**	**762**
**Total**	**48**	**37**	**87**	**69**	**33**	**30**	**67**	**38**	**58**	**58**	**77**	**41**	**163**	**76**	**171**	**84**	**704**	**433**	**1137**

Table S4: The number of singletons and clustered RGAs affected by CNV across 563 *R* genes.

	In cluster				Singleton							
RGAs	Del	Dup	Both	**Sum**	Del	Dup	Both	**Sum**	**Sum of Del**	**Sum of Dup**	**Sum of Both**	**Total**
CN	5	2	2	**9**	2	0	0	**2**	**7**	**2**	**2**	**11**
CNL	12	7	3	**22**	7	3	0	**10**	**19**	**10**	**3**	**32**
NBS	12	3	2	**17**	2	2	0	**4**	**14**	**5**	**2**	**21**
NL	11	4	2	**17**	8	4	4	**16**	**19**	**8**	**6**	**33**
OTHER	2	1	0	**3**	2	1	0	**3**	**4**	**2**	**0**	**6**
TN	9	1	0	**10**	1	0	0	**1**	**10**	**1**	**0**	**11**
TNL	18	8	3	**29**	2	0	1	**3**	**20**	**8**	**4**	**32**
TX	10	4	0	**14**	3	1	0	**4**	**13**	**5**	**0**	**18**
**Sum of NBS-LRR and TX**	**79**	**30**	**12**	**121**	**27**	**11**	**5**	**43**	**106**	**41**	**17**	**164**
RLK	71	31	8	**110**	89	101	19	**209**	**160**	**132**	**27**	**319**
RLP	30	14	9	**53**	14	9	4	**27**	**44**	**23**	**13**	**80**
**Sum of TM-LRR**	**101**	**45**	**17**	**163**	**103**	**110**	**23**	**236**	**204**	**155**	**40**	**399**
**Total**	**180**	**75**	**29**	**284**	**130**	**121**	**28**	**279**	**310**	**196**	**57**	**563**

Table S5: Distribution and number of the singletons and clustered *R* genes affected by CNV across the chromosomes.

Chromosome	*R* genes number	Clustered *R* genes number	Clusters number	Singleton *R* genes number	Clustered *R* genes affected by CNV	Singletons *R* genes affected by CNV
A01	66	24	11	42	6	16
A02	75	40	12	35	22	13
A03	87	28	14	59	12	22
A04	60	31	13	29	8	5
A05	52	22	9	30	8	8
A06	104	50	20	54	21	24
A07	90	41	17	49	12	8
A08	85	40	16	45	16	12
A09	140	63	25	77	35	39
A10	41	15	6	26	1	4
C01	107	55	23	52	10	14
C02	115	58	19	57	26	15
C03	149	49	19	100	21	27
C04	83	33	16	50	5	13
C05	91	34	13	57	5	10
C06	118	75	24	43	29	7
C07	75	31	9	44	2	4
C08	95	53	19	42	28	5
C09	135	51	21	84	17	33
**Sum**	**1,768**	**793**	**306**	**975**	**284**	**279**

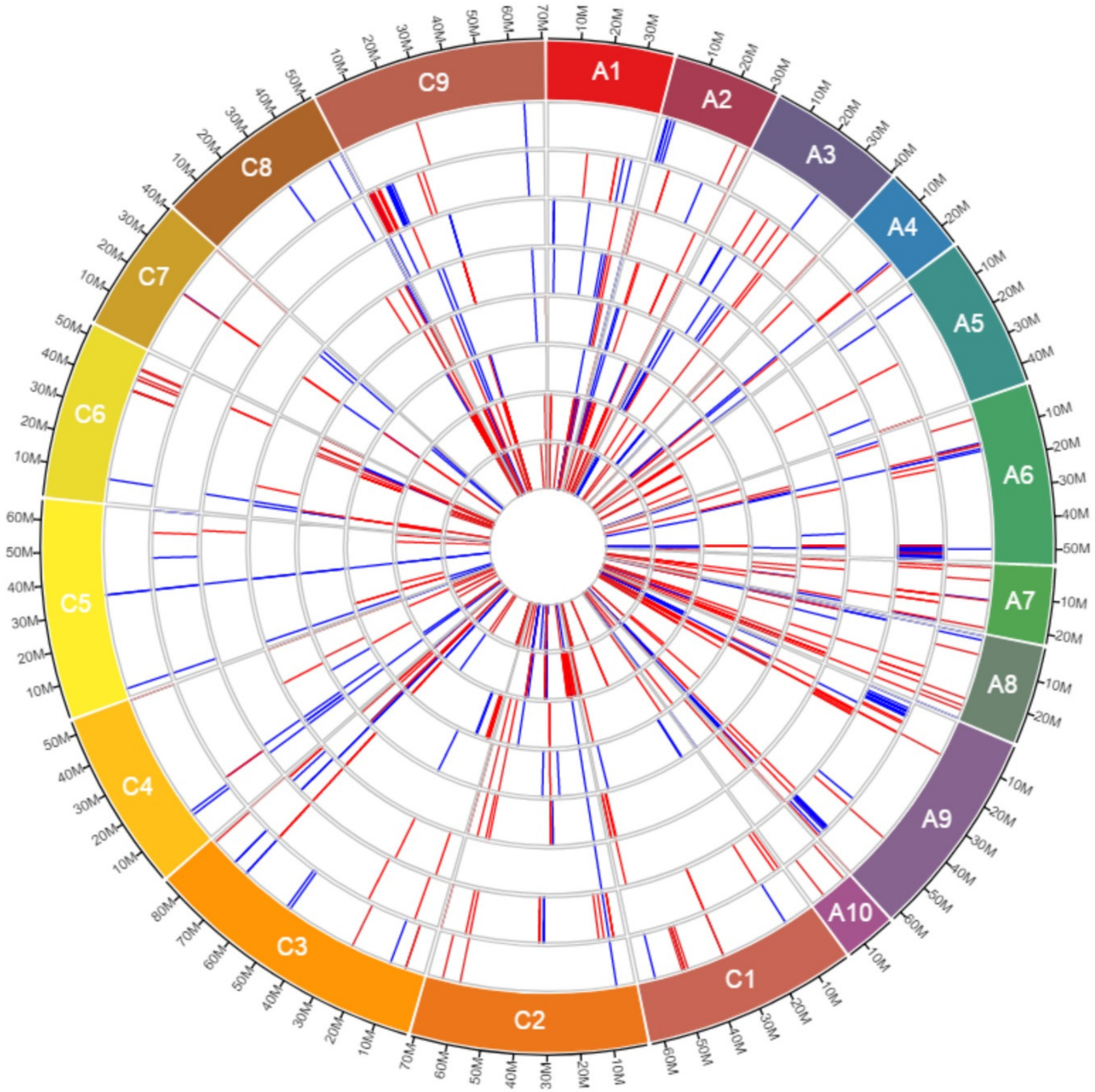
A1
A2
A3
A4
A5
A6
A7
A8
A9
A10
C1
C2
C3
C4
C5
C6
C7
C8
C9

Figure S1: The position of CNV events (red and blue lines represents deletions and duplications, respectively) across the chromosomes of eight *B. napus* lines. The tracks from outer to inner show chromosomes, Ascona, English Giant, Hansen × Gaspard, Milena, Pacific, Pirola, Tina and Wilhelsburger.
